# Targeting Ferroptosis/Nrf2 Pathway Ameliorates AlCl_3_-Induced Alzheimer’s Disease in Rats: Neuroprotective Effect of Morin Hydrate, Zeolite Clinoptilolite, and Physical Plus Mental Activities

**DOI:** 10.3390/ijms26031260

**Published:** 2025-01-31

**Authors:** Karema Abu-Elfotuh, Yasmin Mahran, Walaa Bayoumie El Gazzar, Heba S. Youssef, Ahmed M. E. Hamdan, Tariq Mohammed Albalawi, Maha Alsunbul, Reem ALQahtani, Asmaa A. Mohammed

**Affiliations:** 1 Clinical Pharmacy Department, Faculty of Pharmacy (Girls), Al-Azhar University, Cairo 11651, Egypt; karimasoliman.pharmg@azhar.edu.eg; 2 Research Department, Natural and Health Sciences Research Center, Princess Nourah bint Abdulrahman University, P.O. Box 84428, Riyadh 11671, Saudi Arabia; 3 Department of Anatomy, Physiology and Biochemistry, Faculty of Medicine, The Hashemite University, Zarqa 13133, Jordan; wallagazzar@hu.edu.jo; 4 Department of Medical Biochemistry and Molecular Biology, Faculty of Medicine, Benha University, Qalyubia 13518, Egypt; 5 Department of Physiology, Faculty of Medicine, Benha University, Qalyubia 13518, Egypt; heba.youssef@fmed.bu.edu.eg; 6 Department of Pharmacy Practice, Faculty of Pharmacy, University of Tabuk, Tabuk 71491, Saudi Arabia; 7 Faculty of Pharmacy, University of Tabuk, Tabuk 71491, Saudi Arabia; tareqov991100@gmail.com; 8 Department of Pharmaceutical Sciences, College of Pharmacy, Princess Nourah bint Abdulrahman University, P.O. Box 84428, Riyadh 11671, Saudi Arabia; maalsonbel@pnu.edu.sa (M.A.); rgalqahtani@pnu.edu.sa (R.A.); 9 Pharmacology and Toxicology Department, Faculty of Pharmacy (Girls), Al Azhar University, Cairo 11651, Egypt; asmaamahmoud.52@azhar.edu.eg

**Keywords:** Alzheimer’s disease, β-amyloid, neurotoxicity, morin hydrate, physical and mental activities, tau, zeolite clinoptilolite

## Abstract

Alzheimer’s disease (AD) is a significant health challenge in the 21st century. In spite of the approval of many new disease-modifying therapies for AD, the clinical advantages of these new treatments are less certain. Aim: This investigation was intended to determine the potential neuroprotective impact of morin hydrate (MH), zeolite clinoptilolite (ZC), and/or physical and mental activities (PhM) on an aluminum chloride (AlCl_3_)-induced AD rat model. Methods: Male Sprague Dawley rats were randomly allocated into seven groups. Group I was the control group. Groups II–VII were treated with AlCl_3_ for 5 weeks. Groups III–VII were tested for the effects of MH, ZC, and/or PhM. Biochemical, brain histopathological, and behavioral studies were performed. Results: PhM, MH, and ZC combined therapy exhibited a significant neuroprotective effect demonstrated by corrected catecholamines and tau and β-amyloid levels, as well as the antioxidant and anti-ferroptotic effects probably through Nrf2/HO-1/GPX4 and ACSL4 signaling pathways. In addition, combined therapy counteracted the inflammatory responses through modulating the TLR4/NF-κβ/NLRP3 inflammasome expression. Moreover, combined therapy groups showed the maximum improvement of both APOE4/LRP1 and Wnt3/β-catenin/GSK-3β signaling expressions. Conclusion: This research highlights the neuroprotective impact of MH and ZC plus PhM against AlCl_3_-induced AD via modulation of Nrf2/HO-1/GPX4, TLR4/NF-κβ/NLRP3, APOE4/LRP1, and Wnt3/β-catenin/GSK-3β signaling pathways. It is the first to point out the inclusion of ferroptosis-Nrf2/inflammasomes cross-talk in the neuroprotection mechanism of MH/ZC against the AlCl_3_-mediated AD model.

## 1. Introduction

Alzheimer’s disease (AD) is a neurodegenerative disorder manifested by β-amyloid (Aβ) extracellular plaques and intracellular tau neurofibrillary tangles [1,2]. Positive or “overt” lesions, Aβ plaques, and tau neurofibrillary tangles are believed to be indicative of the pathophysiology of AD. Alternatively, loss of neurons, synaptic homeostasis, or the integrity of neural networks might be considered as negative (“covert”) aspects associated with AD [3,4].

There is an evidence that aluminum (Al) is a neurotoxic xenobiotic that alters a number of proteins linked to neurotoxicity [5,6,7]. The presence of Al in food, the environment, and daily activities renders exposure to it practically inescapable [8,9]. Aβ oligomer cross-linking and deposition, plaque formation, and oxidative stress have all been demonstrated to be accelerated by Al in hippocampus and cortex of brain [6,10]. Therefore, AD induced in rats by aluminum chloride (AlCl_3_) provides a useful model for investigating AD as well as the possible neuroprotective agents [10,11,12].

It is generally known that inflammation and oxidative damage are unavoidable characteristics linked to AD. Nuclear factor erythroid 2 related factor 2 (Nrf2), a transcriptional factor mediating the cell redox balance, translocates from cytoplasm to nucleus under oxidative stress, resulting in activation of heme oxygenase-1 (HO-1) [13,14,15,16]. Moreover, high and prolonged oxidative stress reduces Nrf2 expression, resulting in the downregulation of antioxidant defenses [17].

In addition, the expression of glutathione peroxidase 4 (GPX4) and cystine transporter solute carrier family 7 member 11 (SLC7A11) are also transcriptionally increased by Nrf2. GPX4 is a critical regulator of ferroptosis, a cell death that is dependent on lipid peroxidation and iron. It is necessary for the conversion of lipid hydroperoxides into non-toxic lipids. Furthermore, the cystine/glutamate antiporter system xc- can be disrupted to induce ferroptosis. This might be seen as the accumulation of lipid peroxides and induction of ferroptosis, which is the outcome of the reduction in GSH levels and GPX4 activity by inhibiting SLC7A11 [18,19]. Therefore, it has been observed that the activation of Nrf2 signalling safeguards cells from ferroptosis in a variety of chronic illnesses [20,21]. Furthermore, Acyl-CoA synthetase long chain family member 4 (ACSL4), a key enzyme in production of lipid peroxides, has been determined to be a critical determinant of ferroptosis sensitivity [22,23]. Thus, evaluating its expression provides evidence for ferroptosis as a unique cell death process in the development of AD.

Activation of caspase-1 is nsoteworthy because it promotes the maturation of proinflammatory cytokines IL-18 and interleukin-1β (IL-1β) and cleaves gasdermin D (GSDMD) to cause pyroptosis. Refs. [24,25,26]. Moreover, it has been discovered that pyrin domain (PYD)-containing protein 3 (NLRP3) inflammasomes are mechanistically implicated in the pathophysiology of AD [27,28]; thus, understanding the various mechanisms that contribute to NLRP3 activation during AD is crucial. Indeed, both priming (Signal 1) and protein complex formation (Signal 2) are necessary for NLRP3 inflammasome activation via nuclear factor-κB (NF-κB)-dependent pathway [26,29]. Therefore, toll-like receptor 4 (TLR4) and NLRP3 inflammasomes are vital molecules in the pathological context of AD through regulating neuroinflammation.

In addition, Wnt/β-catenin signaling activation offers neuroprotective advantages by reducing Aβ accumulation and tau hyperphosphorylation [30,31,32]. Without Wnt ligands, ß-catenin is phosphorylated by glycogen synthase kinase-3β (GSK-3β), causing it to deteriorate [32,33]. Although the Wnt/β-catenin trajectory is mostly impeded by GSK-3β, the canonical Wnt signaling trajectory activates GSK-3β activity, which in turn stabilizes β-catenin and activates transcription of Wnt-targeted genes [34,35]. Therefore, modification of the Wnt 3/β-catenin/GSK-3β trajectory has been suggested to be linked to AD’s etiology. Activating Wnt 3/β-catenin/GSK-3β pathway is therefore thought to be a promising option for AD therapeutics. Other vital players in the pathological context of AD are Apolipoprotein E4 (ApoE4) and LDL receptor-related protein 1 (LRP1), which is considered the best indicator of late-onset AD [36,37,38,39]. Preclinical studies have recommended that the apoE4-LRP1 axis may promote the harmful impacts of apoE4 [40].

Experimental and clinical research has demonstrated that consuming flavonoids is associated with better cognitive function [41]. A naturally occurring flavonoid belonging to the Moraceae family, morin hydrate (MH) has anti-inflammatory and antioxidant properties that have been demonstrated to be useful in treating some neurological illnesses. Refs. [12,42,43], via reduction in both Aβ plaques and hyperphosphorylated tau protein in neurons [12,44,45]. MH showed its pharmacological properties through modulation of various cellular signaling pathways in various diseases [43], while studies investigating MH effect on the crucial signalling pathways contributed to the pathophysiology of AD are limited.

Zeolites are a class of hundreds of members of microporous minerals that are distinguished by their ability to exchange ions. The zeolite clinoptilolite (ZC), which is naturally occurring, is the most extensively investigated and present. The modification as micronization of ZC (M-ZC) enables the enhancement of its advantages in preclinical and clinical scenarios. ZC is a potent immunomodulatory, anti-inflammatory, antioxidant, and detoxifying agent. It has the ability to adsorb and eliminate toxic compounds from the human gastrointestinal system, such as ammonia, heavy metals, or other minute molecules [46]. Although there is a proof for the influence of ZC on AD pathology, it has been hypothesized that ZC acts indirectly at the level of the CNS given the existence of the gut–brain link [47,48,49]. However, it remains an unknown domain, necessitating the development of numerous new and comprehensive research studies to determine its possible neuroprotective function and the molecular mechanisms that underlie its beneficial effects.

Physical and mental activities (PhM) reduce the risk of AD by 45% [50]. They have received greater attention as a non-pharmacological therapy against AD. Jiang et al. reported that swimming exercise has a direct impact on the hippocampal expression of neurotrophic factors [51]. In addition, Serra et al. reported that the open field test and walking stimulate cognitive function [52]. In fact, physical inactivity has been recognized as one of the non-genetic risk factors related with AD. By lowering brain Aβ plaques and amyloid angiopathy, cognitive and physical exercises may delay the progression of AD illness and enhance cognitive skills [53]. Rodents have demonstrated comparable cognitive training outcomes [54,55]. However, the cellular signaling and downstream mechanisms underlying these protective effects remain limited.

When considering the convergence of multiple signaling pathways in AD progression and the reliable failures of monotherapies for AD, it is possible that an integrated combined approach will be required to develop multifaceted treatments that may ultimately prove more effective. Consequently, the current study was devised with an animal model of AlCl_3_-induced neurotoxicity to determine the molecular mechanisms that underlie the potential neuroprotective impacts of MH, ZC, and physical and mental activities (PhM), separately or combined, by examining their effects on Nrf2/HO-1/GPX4, a ferroptosis-related gene; ACSL4; TLR4/NF-κβ/NLRP3; APOE4/LRP1; and Wnt 3/β-catenin/GSK-3β signaling pathways. In addition, behavioral and brain histopathological parameters were determined.

## 2. Results

### 2.1. Treatment with MH and/or ZC Combined with PhM Improved the AlCl_3_-Induced Altered Behavioral, Learning, and Memory Parameters

#### 2.1.1. Y-Maze Spontaneous Alternation Test

**Figure 1:**
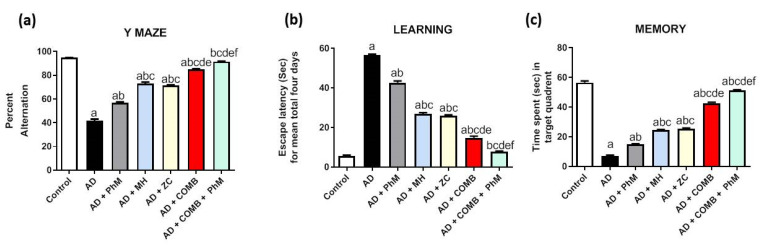
The effects of MH and/or ZC combined with PhM on behavioral, learning, and memory parameter alterations induced by AlCl_3_: (**a**) Y maze, (**b**) learning, and (**c**) memory. The data are displayed as means ± SD (*n* = 6) using one-way ANOVA and then Tukey–Kramer as the post hoc test. Significance a, with respect to the control group; significance b, with respect to the AD group; significance c, with respect to the AD + PhM group; significance d, with respect to the AD + MH group; significance e, with respect to the AD + ZC group; significance f, with respect to the AD + COMB group. Significance: *p* < 0.05. AD, Alzheimer’s disease; COMB, combination therapy; MH, morin hydrate; PhM, physical and mental activity; ZC, zeolite clinoptilolite.

Rats intoxicated with AlCl_3_ showed a discernible 56% decrease in SAP% during Y-Maze spontaneous alternation in proportion to the control group. However, as opposed to AD rats, this lessening was greatly improved in PhM and MH and ZC administration by 1.4, 1.8, and 1.7, respectively. In a similar vein, COMB therapy provided extra advantages. However, COMB + PhM showed the greatest amelioration of the spatial working memory deficit of AD rats by 2.2 when compared with each individual treatment (Figure 1a).

#### 2.1.2. Morris Water Maze Test (MWM)

MWM test outcomes demonstrated that rats given AlCl_3_ had a significant impairment in their spatial learning, as indicated by the fact that the rats’ mean escape latency was dramatically greater by ten times (Figure 1b) and that they spent an 88% shorter period of time in the target quadrant than the control group (Figure 1c). The administration of MH and ZC together with AlCl_3_ ameliorated the deficits in spatial memory and learning. The decline in the average escape latency throughout the four days served as evidence of this (25, 52, and 54%), as well as the rise in the duration spent in the aim quadrant (2.1-, 3.5-, and 3.6-fold) in contrast to the AlCl_3_ control group. Remarkably, when compared with each individual therapy, the COMB + PhM-treated group had the best outcomes in terms of escape latency and the largest amount of time spent in the target quadrant. Compared with the AD group, the escape latency was 86% lower in the AD + PhM + COMB group. In contrast to the AD group, spending more time in the target quadrant levels rose by a factor of 7.3.

### 2.2. Treatment with MH and/or ZC Combined with PhM Counteracted the AlCl_3_-Induced Histopathological Changes in Brain Tissues

**Figure 2:**
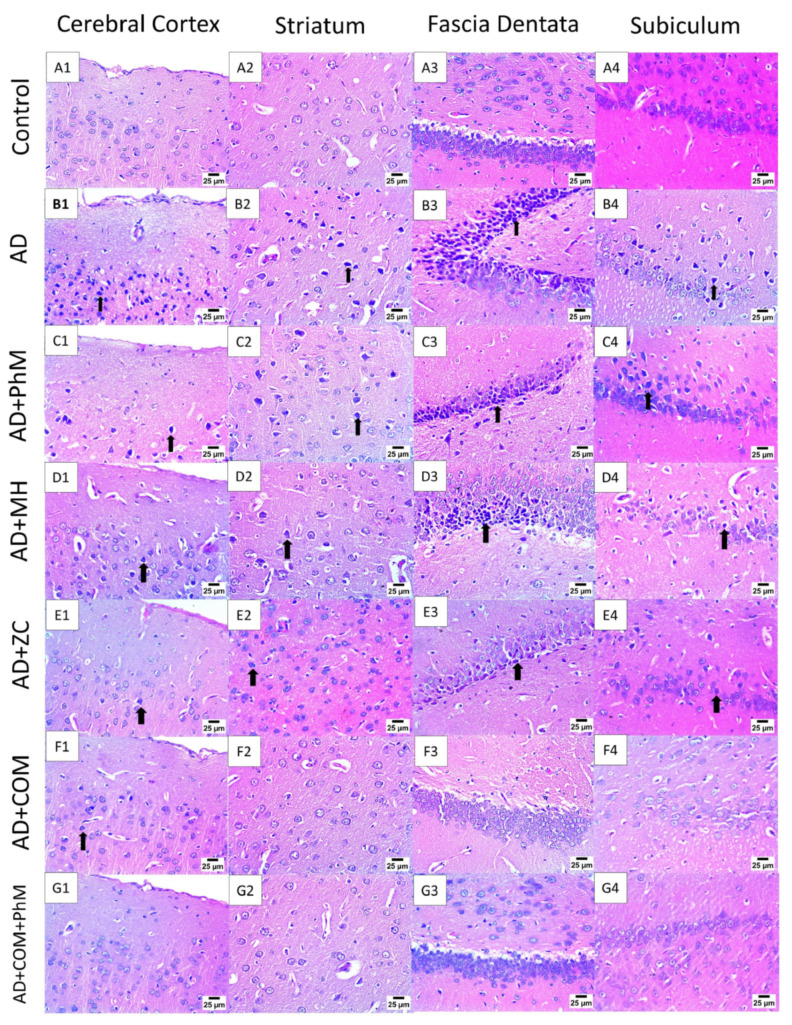
Photomicrographs of brain sections stained by Haematoxylin and Eosin (scale bar 25 μm). Brain sections of the control group exhibited no histopathological alterations in the presented brain areas: the cerebral cortex, striatum, subiculum, and fascia. In the AD group, there were a great number of shrunken and degenerated neurons with severe nuclear pyknosis in the cerebral cortex, striatum, and subiculum (arrow) and severe nuclear pyknosis in neurons of the fascia dentata (arrow). In the AD + PhM group, there was a moderate number of shrunken and degenerated neurons with nuclear pyknosis in the cerebral cortex and striatum (arrow) and severe nuclear pyknosis in the fascia dentata and subiculum (arrow). In the AD + MH, a few shrunken and degenerated neurons showed nuclear pyknosis in the cerebral cortex and striatum (arrow) and moderate nuclear pyknosis in the fascia dentata and subiculum (arrow). In the AD + ZC group, there was mild nuclear pyknosis in neurons of the cerebral cortex, striatum, and subiculum (arrow) and moderate nuclear pyknosis in neurons of the fascia dentata (arrow). In the AD + COMB, there was mild nuclear pyknosis in neurons of the cerebral cortex (arrow) and normal histological structure of neurons in the striatum, fascia dentata, and subiculum. Finally, there were no histopathological findings in the AD + COMB + PMA group. AD, Alzheimer’s disease; COMB, combination therapy; MH, morin hydrate; PhM, physical and mental activity; ZC, zeolite clinoptilolite.

As presented in Figure 2, a light microscopic analysis of the AlCl_3_-treated group demonstrated that the entire brain structure was impacted since the cerebral cortex’s neurons showed signs of nuclear necrosis and degeneration. Additionally, there was nuclear pyknosis and degeneration accompanied by blood vessel congestion in the neurons of the fascia dentate, substantia nigra and striatum, and pyramidal cells of the hippocampus. Concurrent PhM showed a moderated number of shrunken and degenerated neurons with nuclear pyknosis in the cerebral cortex and striatum and severe nuclear pyknosis in the fascia dentata and subiculum. As well, the administration of MH and Zc to AlCl_3_-treated rats showed mild to few numbers of shrunken and degenerated neurons with nuclear pyknosis in the cerebral cortex and striatum with moderate nuclear pyknosis in the fascia dentata and subiculum. Notably, combination therapy showed mild nuclear pyknosis in neurons of the cerebral cortex with typical neuronal histological structure in the striatum, fascia dentata, and subiculum. Furthermore, combination treatment with PhM revealed that the neurons in the subiculum, striatum, fascia dentata, and cerebral cortex had normal histological structures.

### 2.3. MH and/or ZC Combined with PhM Improved the AlCl_3_-Induced Changes in Neurotransmitter Brain Levels (DA, 5-HT, NE, and ACHE)

DA, 5-HT, and NE were diminished by 81%, 84%, and 76%, respectively, in the AlCl_3_-intoxicated group, as well as a six-fold elevation in ACHE levels contrary to the control group. Conversely, PhM, as well as concurrent administration of MH or ZC with AlCl_3_, significantly increased the levels of neurotransmitters and avoided the higher levels of ACHE produced by AlCl_3_ as opposed to the AlCl_3_ control group. PhM augmented DA, 5-HT, and NE levels by 1.7-, 2.2-, and 1.4-fold, respectively. In addition, MH augmented their levels by 2.9-, 3.1-, and 2.2-fold, respectively, and for ZC, the levels were stimulated by 2.6-, 3.8-, and 2.5-fold, respectively. Regarding ACHE, PhM reduced its level by 11%. MH reduced its level by 36%, and ZC suppressed it by 49%. In a similar vein, COMB treatment provided extra advantages. However, as compared with each individual therapy, PhM in addition to COMB therapy substantially exhibited the highest protective effects against AlCl_3_-induced alterations in neurotransmitter levels. As opposed to the AD group, DA, 5-HT, and NE levels in the AD + PhM + COMB group improved by 4.3-, 5.8-, and 3.6-fold, respectively. When comparing ACHE levels with the AD group, there was an 80% drop (Table 1).

### 2.4. Treatment with MH and/or ZC Combined with PhM Ameliorated the AlCl_3_-Induced Induction of Oxidative Stress Biomarkers and Nrf2/HO-1 Signaling Pathway

#### 2.4.1. The Impact of MH and/or ZC Therapy Combined with PhM on the AlCl_3_-Induced Altered Nrf2/HO-1 Signaling Pathway

**Figure 3:**
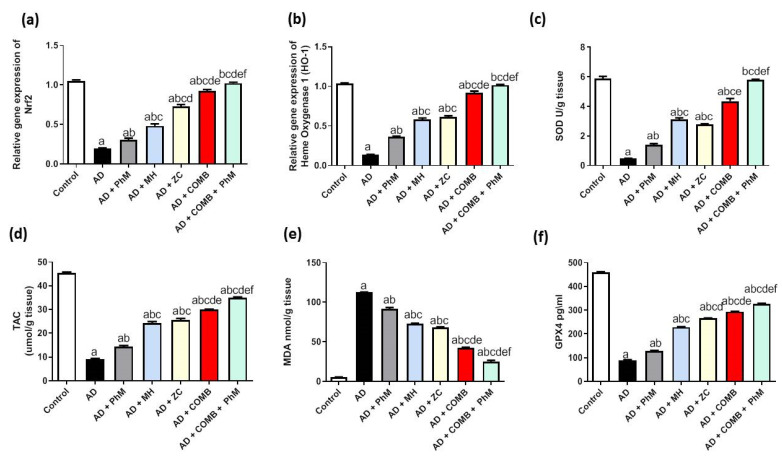
The effect of MH and/or ZC combined with PhM on oxidative stress marker changes induced by AlCl_3_: (**a**) Nrf2, (**b**) HO-1, (**c**) SOD, (**d**) TAC, (**e**) MDA, and (**f**) GPX4. The data are displayed as means ± SD (*n* = 6) using one-way ANOVA and then Tukey–Kramer as the post hoc test. Significance a, with respect to the control group; significance b, with respect to the AD group; significance c, with respect to the AD + PhM group; significance d, with respect to the AD + MH group; significance e, with respect to the AD + ZC group; significance f; with respect to the AD + COMB group. Significance: *p* < 0.05. AD, Alzheimer’s disease; COMB, combination therapy; GPX4, glutathione peroxidase 4; HO-1, heme-oxygenase-1; MDA, malondialdehyde; MH, morin hydrate; Nrf2, nuclear factor erythroid 2–related factor 2; PhM, physical and mental activity; SOD, superoxide dismutase; TAC, total antioxidant capacity; ZC, zeolite clinoptilolite.

Rats treated with AlCl_3_ exhibited a significant drop in Nrf2 and HO-1 gene expressions (82% and 87%, respectively), as opposed to the control group (Figure 3a,b). on the contrary, PhM, as well as concurrent administration of MH or ZC with AlCl_3_, significantly raised Nrf2/HO-1 signaling by boosting the mRNA expressions of Nrf2 by 1.6-, 2.5-, and 3.7-fold, respectively, and HO-1 by 2.7-, 4.3-, and 4.6-fold, respectively, thus resulting in neuroprotective effects in contrast to the AD group. Similarly, COMB therapy provided extra advantages. However, as compared with each therapy alone, PhM added to COMB therapy substantially exhibited the most potent protective effects against oxidative stress induced by AlCl_3_. Nrf2/HO-1 signaling levels were enhanced in the AD + PhM + COMB group as opposed to the AD group by 5.2- and 7.5-fold, respectively.

#### 2.4.2. The Impacts of MH and/or ZC Therapy Combined with PhM on the AlCl_3_-Induced Alterations in SOD, TAC, and MDA and GPX4 Levels

AlCl_3_ was sufficient to upset the balance between oxidants and antioxidants, as seen in Figure 3c–e. When AlCl_3_ was administered to the AD group, the levels of SOD and TAC were significantly suppressed by 92% and 80%, respectively. This led to a subsequent rise of MDA levels by 20.6 times, as opposed to the control group. When compared with the AD group, PhM and concurrent administration of MH or ZC with AlCl_3_ dramatically elevated the concentrations of SOD and TAC while also ameliorating heightened levels of MDA. SOD and TAC levels were increased by 2.9 and 1.6 times, respectively, by PhM. Additionally, MH elevated levels of SOD and TAC by 6.4- and 2.7-fold, respectively, whereas ZC increased them by 5.7- and 2.8-fold, respectively. PhM lowered the level of MDA by 19%. MH decreased its level by 35%, whereas ZC inhibited it by 39%. In a similar vein, COMB treatment provided extra advantages. However, as compared with each therapy alone, PhM added to COMB therapy substantially had the strongest protective effects against oxidative stress caused by AlCl_3_. SOD and TAC levels were enhanced in the AD + PhM + COMB group in contrast to the AD group by 11.8 and 3.8 times, respectively. MDA concentrations were 78% lower in the AD group.

In addition, the brain GPX4 content was significantly reduced by 80% in rats given AlCl_3_ as opposed to the control group (Figure 3f). Conversely, PhM, as well as concurrent administration of MH or ZC with AlCl_3_, significantly raised the GPX4 brain content by 140%, 250%, and 290%, respectively, resulting in neuroprotective effects in contrast to the AD group. Similarly, COMB therapy provided extra advantages. However, as compared with each therapy alone, PhM added to COMB therapy substantially had the strongest protective effects against oxidative stress caused by AlCl_3_. As opposed to the AD group, GPX4 levels in the AD + PhM + COMB group improved by 360%.

### 2.5. Treatment with MH and/or ZC Combined with PhM Counteracted the AlCl_3_-Induced Inductions in Inflammatory Pathways

#### 2.5.1. The Impact of MH and/or ZC Therapy Combined with PhM on TLR4/NF-κB/NLRP3/caspase-1 Signaling

**Figure 4:**
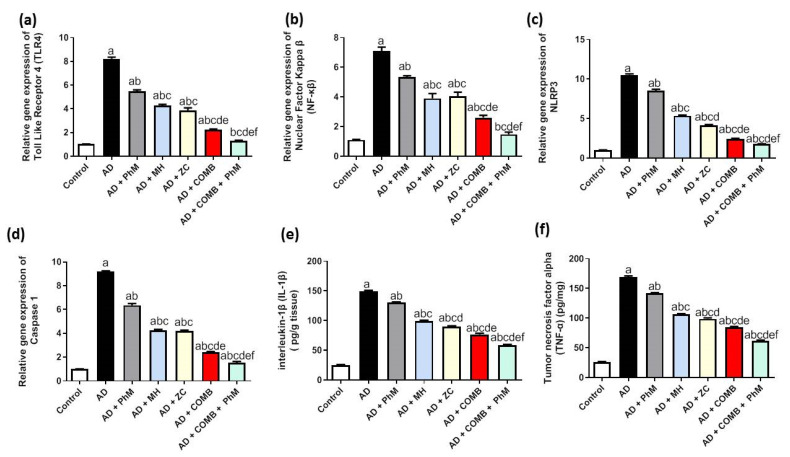
The effects of MH and/or ZC combined with PhM on inflammatory marker changes induced by AlCl_3_: (**a**) TLR4, (**b**) NF-κB, (**c**) NLRP3, (**d**) Caspase-1, (**e**) IL-1β, and (**f**) TNF-α. The data are displayed as means ± SD (*n* = 6) using one-way ANOVA and then Tukey–Kramer as the post hoc test. Significance a, with respect to the control group; significance b, with respect to the AD group; significance c, with respect to the AD + PhM group; significance d, with respect to the AD + MH group; significance e, with respect to the AD + ZC group; significance f, with respect to the AD + COMB group. Significance: *p* < 0.05. AD, Alzheimer’s disease; COMB, combination therapy; IL-1β, Interleukin-1 beta; MH, morin hydrate; NF-κB, nuclear factor-κB; NLRP3, pyrin domain–containing-3; PhM, physical and mental activity; TLR4, Toll-like receptor 4; TNF-α, tumor necrosis factor; ZC, zeolite clinoptilolite.

The activation of the TLR4 signaling pathway by AlCl_3_ therapy was shown by RT-qPCR analysis, as seen in Figure 4a–d. The mRNA expression of TLR4, NF-κB, NLRP3, and caspase-1 was significantly upregulated by 8.1-, 6.4-, 10.3-, and 9-fold, respectively, in comparison with the control group. Conversely, PhM, as well as concomitant administration of MH or ZC with AlCl_3_, significantly decreased TLR4 by 33%, 48%, and 53%; NF-κB by 25%, 45%, and 43%; NLRP3 by 19%, 49%, and 60%; and caspase-1 mRNA expression by 31%, 54%, and 55%, respectively, thus resulting in neuroprotective effects in contrast to the AD group. Similarly, COMB therapy provided extra advantages. However, as compared with each therapy alone, PhM added to COMB therapy considerably demonstrated the highest protective effects against AlCl_3_-induced inflammation. TLR4, NF-κB, NLRP3, and caspase-1 mRNA expression were all increased in the AD + PhM + COMB group as opposed to the AD group by 84%, 79%, 83%, and 83%, respectively.

#### 2.5.2. The Impact of PhM Treatment Combined with MH and ZC Therapy on Pro-Inflammatory Cytokines, IL-1β and TNF-α

As demonstrated in Figure 4e,f, AlCl_3_ treatment significantly increased the levels of pro-inflammatory cytokines (TNF-α and IL-1 β) by roughly 590 and 650 times, respectively, in contrast to the control group. Conversely, PhM and concomitant administration of MH or ZC with AlCl_3_ significantly decreased the IL-1 β by 13%, 33%, and 40% and TNF-α by 16% and 42%, respectively, thus resulting in neuroprotective effects as compared with the AD group. Likewise, using COMB treatment revealed further advantages. However, as compared with each therapy alone, PhM added to COMB therapy considerably demonstrated the highest protective effects against AlCl_3_-induced inflammation. In contrast to the AD group, there was a greater than 61% and 64% improvement in IL-1 β and TNF-α signaling levels in the AD + PhM + COMB group.

### 2.6. MH and/or ZC Therapy Combined with PhM Improved the AlCl_3_-Induced Induction of Apoptosis Biomarkers, Bax and Bcl-2

**Figure 5:**
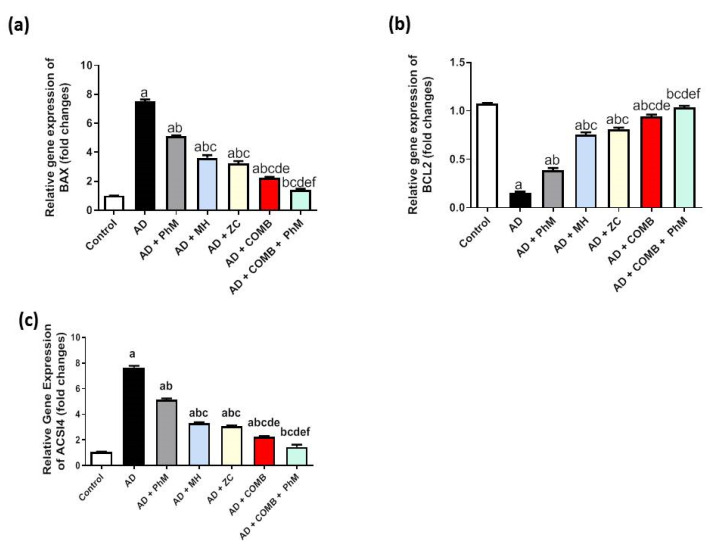
The effect of MH and/or ZC combined with PhM on apoptosis and ferroptosis marker changes induced by AlCl_3_: (**a**) Bax, (**b**) Bcl-2, and (**c**) ACSL4. The data are displayed as means ± SD (*n* = 6) using one-way ANOVA and then Tukey–Kramer as the post hoc test. Significance a, with respect to the control group; significance b, with respect to the AD group; significance c, with respect to the AD + PhM group; significance d, with respect to the AD + MH group; significance e, with respect to the AD + ZC group; significance f, with respect to the AD + COMB group. Significance: *p* < 0.05. ACSL4, Acyl-CoA Synthetase Long Chain Family Member 4; AD, Alzheimer’s disease; Bax, B-cell lymphoma protein 2 (*Bcl-2*)-connected X protein; Bcl-2, B-cell lymphoma protein 2; COMB, combination therapy; MH, morin hydrate; PhM, physical and mental activity; ZC, zeolite clinoptilolite.

Rats treated with AlCl_3_ had a considerable rise (by 7.4-fold) in Bax mRNA expression and a decrease (by 0.9-fold) in Bcl-2 mRNA expression as opposed to the control group, as demonstrated by RT-qPCR analysis, as exhibited in Figure 5a,b. As opposed to the AD group, PhM and the concurrent administration of MH or ZC with AlCl_3_ demonstrated antiapoptotic effects, as evidenced by the upsurge in Bcl-2 mRNA expression by 2.6-, 5-, and 5.4-fold, respectively, and significant decreases in Bax mRNA expression by 32%, 52%, and 57%, respectively. In a similar vein, COMB therapy provided extra advantages. However, as compared with each individual therapy, PhM in addition to COMB therapy considerably had the strongest protective effects against apoptosis caused by AlCl_3_. The mRNA expression of these parameters reverted to baseline in the AD + PhM + COMB group, exhibiting a considerable drop in Bax and an elevation in Bcl2 mRNA expression of 81% and 6.9-fold, respectively, in contrast to the AD group.

### 2.7. Modulatory Effect of MH and/or ZC Combined with PhM on the Ferroptosis Marker ACSL4

AlCl_3_ treatment dramatically increased ACSL4 mRNA expression by almost 7.2 times relative to the control group, as seen in Figure 5c. However, compared with the AD group, PhM and concurrent administration of MH or ZC with AlCl_3_ dramatically reduced ACSL4 signaling by downregulating ACSL4 mRNA expressions by 32%, 56%, and 59%, respectively. This had anti-ferroptotic effects. In a similar vein, COMB therapy provided extra advantages. However, as compared with each individual therapy, PhM added to COMB therapy substantially had the strongest protective effects against cognitive decline brought on by AlCl_3_. In contrast to the AD group, ACSL4 signaling levels in the AD + PhM + COMB group improved by 81%.

### 2.8. Treatment with MH and/or ZC Combined with PhM Improved the AlCl_3_-Induced Changes in BDNF and the Wnt 3/β-catenin/GSK-3β Signaling Pathway

**Figure 6:**
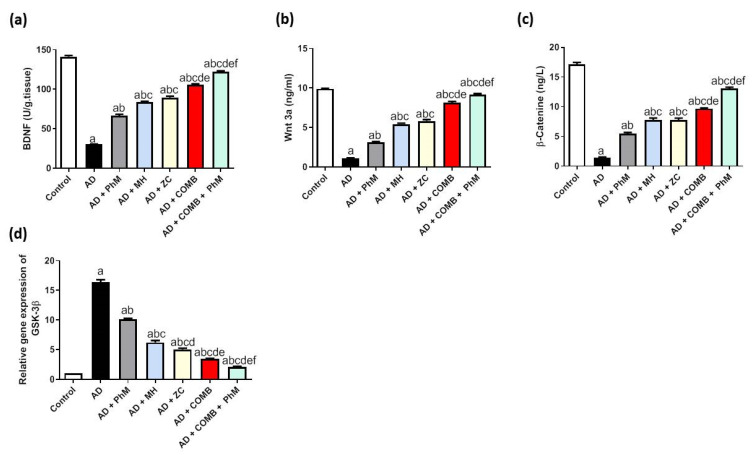
The effect of MH and/or ZC combined with PhM on AlCl_3_-induced changes in BDNF and the Wnt 3/β-catenin/GSK-3β signaling pathway, Wnt3a/β-Catenin. (**a**) BDNF, (**b**) Wnt3a, (**c**) β-Catenin, and (**d**) GSK-3β. The data are displayed as means ± SD (*n* = 6) using one-way ANOVA and then Tukey–Kramer as the post hoc test. Significance a, with respect to the control group; significance b, with respect to the AD group; significance c, with respect to the AD + PhM group; significance d, with respect to the AD + MH group; significance e, with respect to the AD + ZC group; significance f, with respect to the AD + COMB group. Significance: *p* < 0.05. AD, Alzheimer’s disease; BDNF, brain-derived neuroprotective factor; COMB, combination therapy; GSK-3β, glycogen synthase kinase-3β; MH, morin hydrate; PhM, physical and mental activity; ZC, zeolite clinoptilolite.

**Figure 7:**
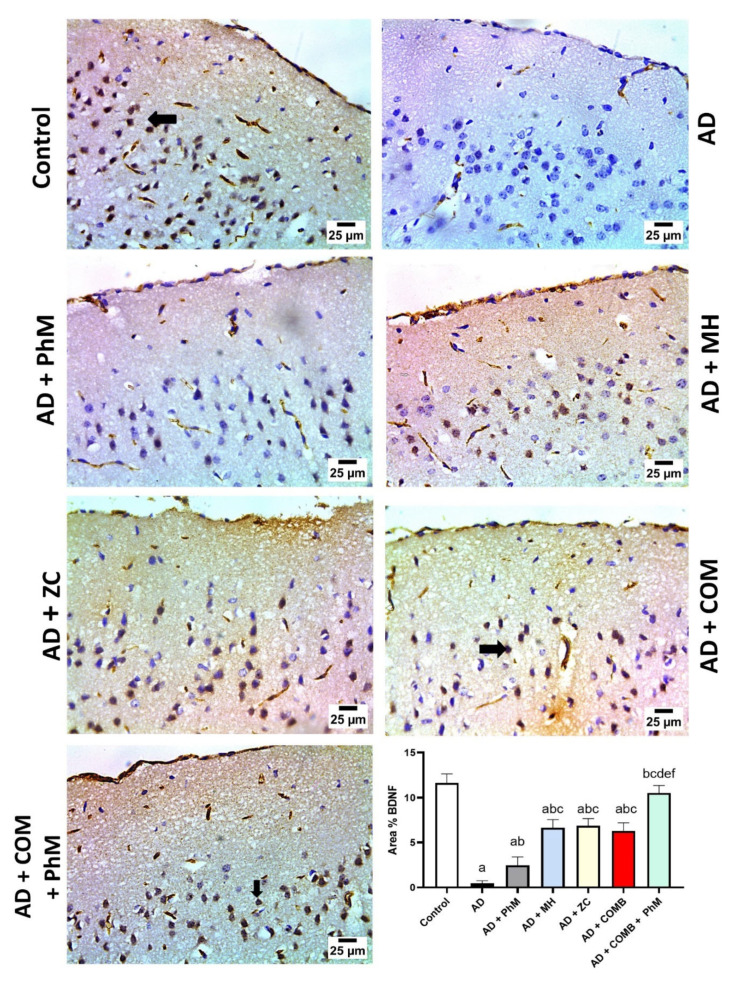
Photomicrographs representing the anti-BDNF antibody-stained brain sections from experimental groups. The control group exhibited severe positive expression for BDNF in neurons of the cerebral cortex. The AD group demonstrated negative expression for BDNF in neurons of the cerebral cortex. The AD + PhM group showed mild positive expression for BDNF in neurons of the cerebral cortex. The AD + MH, AD + ZC, and AD + COMB groups showed moderate positive expression for BDNF in neurons of the cerebral cortex. In the AD + PhM + COMB group, the photomicrograph shows severe positive expression for BDNF in neurons of the cerebral cortex. The data are displayed as means ± SD (*n* = 6) using one-way ANOVA and then Tukey–Kramer as the post hoc test. Significance a, with respect to the control group; significance b, with respect to the AD group; significance c, with respect to the AD + PhM group; significance d, with respect to the AD + MH group; significance e, with respect to the AD + ZC group; significance f, with respect to the AD + COMB group. Significance: *p* < 0.05. AD, Alzheimer’s disease; COMB, combination therapy; MH, morin hydrate; PhM, physical and mental activity; ZC, zeolite clinoptilolite.

As presented in Figure 6a, the brain content of BDNF was significantly reduced by 79% as a result of AlCl_3_ therapy as opposed to the control group. Conversely, PhM and concurrent administration of MH or ZC with AlCl_3_ significantly raised BDNF by 2.2-, 2.8-, and 3-fold), resulting in neuroprotective effects as opposed to the AD group. In a similar vein, COMB treatment provided extra advantages. However, as compared with each individual therapy, PhM added to COMB therapy considerably exhibited the highest protective benefits. In contrast to the AD group, BDNF levels in the AD + PhM + COMB group improved by 4.1. Also, quantitative analyses of the immunohistochemical expression of the BDNF protein were carried out, and the results are shown in Figure 7, which were closely coincident with the biochemical assay of BDNF. The results showed a severe decline in protein expression following AlCl_3_ treatment in contrast to the control group, while therapy with either MH and/or ZC along with PhM significantly upregulated the immunoexpression of BDNF in brain tissues in contrast to AlCl_3_-treated groups.

In addition, Figure 6b,c show the Wnt3a and β-Catenin brain levels were significantly diminished by 89% and 92%, respectively, as compared with the control group. Conversely, PhM, as well as concurrent administration of MH or ZC with AlCl_3_, significantly raised the brain level of Wnt3a by 2.9-, 5-, and 5.3-fold and β-Catenin by 3.8-, 5.3-, and 5.3-fold, respectively, as opposed to the AD group. Furthermore, as compared with each therapy alone, the combination of PhM with MH and ZC therapy demonstrated the greatest protective benefits against AlCl_3_-induced alterations. In addition, Wnt3a/β-Catenin signaling levels were augmented by 8.4- and 9-fold, respectively, in the AD + PhM + COMB group contrary to the AD group.

The therapy with AlCl_3_ dramatically increased the mRNA expression of GSK3β by about 16.2 times in comparison with the control group, as seen in Figure 6d. In contrast to the AD group, PhM and concurrent administration of MH or ZC with AlCl_3_ dramatically reduce GSK3β signaling by downregulating GSK3β mRNA expressions by 38%, 62%, and 69%, respectively. In a similar vein, COMB therapy provided extra advantages. However, as compared with each individual therapy, PhM added to COMB therapy substantially exhibited the most significant protective effects against cognitive decline caused by AlCl_3_. As opposed to the AD group, GSK3β signaling levels were 87% greater in the AD + PhM + COMB group.

### 2.9. Treatment with MH and/or ZC Combined with PhM Improved the AD Progression Markers APP, Aβ, Tau, and BACE1

**Figure 8:**
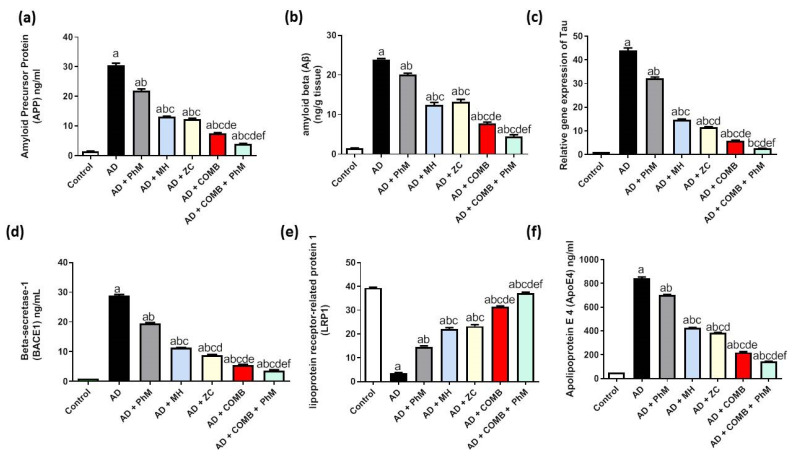
The effect of MH and/or ZC combined with PhM on AlCl_3_-induced AD progression and APOE4/LRP1 signaling pathways. (**a**) APP, (**b**) Aβ, (**c**) Tau, (**d**) BACE1, (**e**) APOE4, and (**f**) LRP1. The data are displayed as means ± SD (*n* = 6) using one-way ANOVA and then Tukey–Kramer as the post hoc test. Significance a, with respect to the control group; significance b, with respect to the AD group; significance c, with respect to the AD + PhM group; significance d, with respect to the AD + MH group; significance e, with respect to the AD + ZC group; significance f, with respect to the AD + COMB group. Significance: *p* < 0.05. AD, Alzheimer’s disease; APOE4, apoliprotein E4; APP, amyloid precursor protein; Aβ, amyloid beta; BACE1, β-secretase enzyme; COMB, combination therapy; LRP1, low-density lipoprotein receptor-related protein 1; MH, morin hydrate; PhM, physical and mental activity; ZC, zeolite clinoptilolite.

Figure 8a–d reveal the enhancement of AD progression markers by AlCl_3_ therapy. This was demonstrated by a significant upsurge of APP, Aβ, Tau, and BACE1 levels by 20.5-, 16.1-, 43-, and 28.1-fold, respectively, contrary to the control group. Conversely, PhM, as well as concurrent administration of MH or ZC with AlCl_3_, significantly decreased APP by 28%, 57%, and 59%; Aβ by 16%, 48%, and 45%; Tau by 27%, 66%, and 74%; and BACE1 by 33%, 61%, and 69%, respectively, thus resulting in neuroprotective effects in contrast to the AD group. Similarly, COMB therapy provided extra advantages. However, as opposed to each individual therapy, PhM added to COMB therapy considerably exhibited the highest protective effects against AlCl_3_-induced neuronal injury. APP, Aβ, Tau, and BACE1 levels were improved in the AD + PhM + COMB group by 87%, 81%, 94%, and 87%, respectively, in contrast to the AD group.

### 2.10. Modulatory Effect of MH and/or ZC Combined with PhM on the APOE4/LRP1 Signaling Pathway

As seen in Figure 8e,f, AlCl_3_ treatment resulted in a significant elevation in the protein expression of APoE4 by 16.7-fold and an aggravating drop in the amount of LRP1 protein expression by 91%. On the other hand, as opposed to rats receiving AlCl_3_ alone, PhM, as well as concurrent administration of MH or ZC with AlCl_3_, dramatically increased LRP1 protein expression by 4.2, 6.3, and 6.6 times, respectively. Similarly, APoE4 protein expression was improved by 17%, 50%, and 54%, respectively. In a similar vein, COMB therapy provided extra advantages. However, as compared with each individual therapy, PhM added to COMB therapy considerably exhibited the highest protective effects against AlCl_3_-induced neuronal injury. LRP1 levels were enhanced, and APoE4 levels declined in the AD + PhM + COMB group as opposed to the AD group by 10.6 times and 83%, respectively.

## 3. Discussion

Even though AD is a public health issue, the available drugs are only symptomatic [56,57]. As a result, there is a significant need for the assistance of natural compounds possessing properties addressing key aspects of AD’s pathophysiology and having significant pharmacological advantages, including immunomodulatory, anti-inflammatory, and antioxidant properties. Additionally, PhM can be used as a novel non-pharmacological approach to increase or maintain cognitive function [53,54,55]. Therefore, in this research, we intended to ascertain the potential neuroprotective impacts of MH, ZC, and PhM activity against AlCl_3_-induced AD by investigating their influence on crucial molecular pathways implicated in the pathophysiology of AD.

Hence, AlCl_3_ is widely used as a model to mimic AD neurotoxicity [58,59], and studies demonstrated altered neurotransmitter metabolism, Aβ, and tau protein aggregation [12,34,59,60,61,62]. Consistent with that, the present study’s findings showed an upsurge in ACHE and a decrease in neurotransmitters following AlCl_3_ administration. In addition, an increase in BACE1 levels that in turn raised the amount of β-amyloid in the brain tissues in the AD group was also demonstrated. Furthermore, the study’s results showed that administering AlCl_3_ caused apoptosis, as shown by the decrease in anti-apoptotic Bcl-2 and the increase in apoptotic Bax expression. It has been reported that both Aβ and AlCl_3_ intoxication cause neuronal cells to undergo apoptosis via a number of different mechanisms [59,63,64,65,66]. In general, the combination of MH and ZC alongside PhM used in this study exerted a neuroenhancement effect supported by improvements in neurotransmitters, BACE1, and β-amyloid levels, as well as decreased tau, Bax expression, and improved behavioral and cognitive functions. However, we intended to carry out a more thorough analysis and look into the molecular basis underlying AlCl_3_-induced AD and the potential neuroprotection of MH and ZC in combination with PhM; therefore, we endeavored to explore the impacts of different signaling pathways in the afforded neuroprotection such as Nrf2/HO-1/GPX4, ferroptosis, TLR4/NF-kB/NLRP3 inflammasomes, Wnt3/β-catenin/GSK-3β, and ApoE4/LRP1.

Data from this research demonstrated that AlCl_3_ administration stimulated an obvious oxidative stress state as demonstrated by the significant oxidant–antioxidant imbalance. Moreover, our experimental results exhibited excessive upregulation of a ferroptosis marker, ACSL4, in brain tissues under oxidative stress, which was manifested by reduced TAC and SOD and elevated MDA levels and was connected to a significant diminishment in Nrf2, HO-1, and GPX4 levels. It has been documented that overexpression of ACSL4 results in the catalysis of various polyunsaturated fatty acid (PUFAs) and the enhancement of lipid hydroperoxide production. In this context, GPX4 is known to transform lipid peroxides (L-OOH) into non-toxic lipids, thereby preventing iron and oxygen-dependent lipid peroxidation [67]. Therefore, PUFAs in the cellular membrane may produce peroxide in the event of GPX4 malfunction, resulting in excessive lipid peroxidation and ferroptosis [23,68]. Therefore, ACSL4 has been reported to address ferroptosis in response to the direct GPX4 inhibition mechanism [69].

In addition, Nrf2 activation has been known for its resistance to the ROS leading to induction of the downstream enzymes HO-1, GPX4, and SOD. Furthermore, by upstream controlling Redox homeostasis, inflammasomes activation, mitochondrial activity, and lipid metabolism, Nrf2 signaling is linked in numerous molecular components of ferroptosis [16,19,70,71]. The results in this study found that Nrf2 signaling was downregulated in all rats with high ACSL4 expression, and this might be a contributing factor to the elevated oxidative stress and eventually ferroptosis. Consistent with these results, previous studies have demonstrated significant induction of oxidative stress connected to impaired Nrf2/HO-1 signaling in brain tissue following AlCl_3_ administration [12,72,73]. In line with our results, as regards ferroptosis, Yan et al. (2022) found an elevated expression of ACSL4 in the hippocampal transcriptome of an APP/PS1 mouse model [74]. Also, GPX4 downregulation has been reported in different cognitive impairment mouse models [75,76].

In this current study, the combination of MH and ZC alongside PhM augmented the antioxidant capacity as evidenced by the significant increment in the TAC, SOD, Nrf2, HO-1, and GPX4 levels, and this was interestingly accompanied by lower ACSL4 expression. Our results first point out that induction of Nrf2/HO-1 signaling mediated by the combination therapy might inhibit ferroptosis and contribute to the neuroprotection mechanisms afforded by the MH/ZC/PhM combination therapy. The antioxidant action of MH [12,77,78], ZC [79,80], and PhM [81,82] was demonstrated in previous studies, which further support this result. However, few studies have yet to demonstrate the precise impact of ZC on human redox systems. According to Lamprecht et al. (2015), there was no discernible impact on free radical generation when ZC supplementation was included in sports nutrition [83].

Furthermore, chronic neuroinflammation is one of the pathological hallmarks of AD [84]. NLRP3 is currently the most extensively determined inflammasome that is incorporated in several chronic inflammatory diseases. Increasing studies have identified its crucial part in Aβ-induced inflammation and hence in the pathogenesis of AD [27,85,86,87,88]. However, mechanisms by which extracellular Aβ activates the cytoplasmic NLRP3 inflammasome remain unclear. Additionally, research has demonstrated that Aβ binds to TLR4 on the surface of astrocytes and microglia, activating NF-κB signaling pathways and causing the production of proinflammatory molecules such as IL-1β, IL-6, and TNF-α [89]. It has also been demonstrated that when the transcription factor NF-kB is activated, NLRP3, pro-IL-1β, and pro-IL-18 are produced, which primes the inflammasome [26,90]. Hence, a cross-talk between NLRP3 inflammasome activation and NF-κB has been suggested and has been reported to be regulated by Nrf2/Ho-1 signaling. In this study, our data unambiguously demonstrated the presence of neuroinflammation via NLRP3 inflammasome signaling through the elevation of TLR4/NLRP3/caspase-1 signaling and the release of pro-inflammatory cytokines, IL-1β and TNF-α. These results were consistent with the inflammatory reactions that AlCl_3_ has been shown to cause in rats [12,72,91,92]. Herein, we provide evidence on the obvious anti-inflammatory role of the combination of MH and ZC alongside PhM demonstrated by significant downregulation of the TLR4/NLRP3/caspase-1 signaling pathway as opposed to the AD group. In the same line with our results, morin has been documented to suppress the expression of TLR4/NF-κβ/NLRP3 in previous studies [12,93,94,95]. In addition, Yang et al. (2021) and Hamdan et al. (2021) have exhibited that exercise had an inhibitory impact on the activation of the NLRP3 inflammasome (2022) [12,96]. Clinoptilolite has also been shown to mitigate inflammation by diminishing levels of TNF-α, IL-1β, and NF-κB in liver cell cultures [97]; additionally, it has been demonstrated to amplify and prolong the anti-inflammatory effects of diclofenac sodium [98]. In addition, there are detoxifying and gut-protective effects of ZC, as well as the gut–brain interaction [46]. We cannot rule out the indirect beneficial effect of ZC on the central nervous system, which necessitates additional in-depth research.

In fact, it is generally acknowledged that NF-κB signaling is a critical regulatory pathway that leads to the regulation of oxidative stress and inflammation. Ferroptosis has been connected to the NF-κB signaling pathway, according to certain research. Dimethyl fumarate alleviated neuroinflammation and ferroptosis in this context by inhibiting the NF-κB signaling pathway [99]. In the same vein, the NLRP3 inflammasome is included in ferroptosis activation, and both have been shown to be regulated by Nrf2 signaling. The NLRP3 inflammasome activation was demonstrated to be triggered by the accumulation of intracellular ferrous through the cGAS-STING1 pathway. This process also resulted in oxidative stress, lipid peroxidation, and ferroptosis. Additionally, the ferroptosis inhibitor ferrostatin-1 reduced the production of NLRP3, IL-1β, and caspase-1, while the ferroptosis inducer, Erastin, enhanced the activation of NLRP3 inflammasomes in placental trophoblast cells. Therefore, our study is the first to explore that the ferroptosis–inflammasome cross-talk might have been involved in the neuroprotection afforded by MH and/or ZC against the AlCl_3_-mediated AD model [67,100].

In addition, Wnt-signaling pathways lead significantly to the preservation of appropriate neuronal function in the adult human brain [33,101,102,103]. In canonical Wnt-β-catenin signaling, the sequestration of GSK3β by the Wnt receptor complex permits β-catenin to accumulate and translocate into the nucleus, enhancing the anti-amyloidogenic α-secretase expression and inhibiting BACE1 expression [103,104]. Nevertheless, the in vitro stimulation of rat neurons with Aβ suppresses the Wnt-signaling cycle, resulting in the increase in GSK3β activity, a decrease in β-catenin levels, and a contribution to neuron death [104]. As a result, it is seen as advantageous to suppress GSK-3β in order to avert neurodegeneration and AD [105]. Furthermore, Wnt signaling could lead to the formation of hyperphosphorylated tau clumps, which can cause neuronal damage by triggering apoptosis, oxidative stress, neuroinflammation, and mitochondrial disorder [106,107,108]. Our findings revealed dysfunctional Wnt signaling in the AlCl_3_ administered group as opposed to the control as suggested by the substantial increase in the expression level of GSK-3β associated with the significant diminishment in Wnt3a, the strongest Wnt/β-catenin stimulator, and β-catenin levels. Interestingly, the amalgamation of MH and ZC alongside PhM in this study significantly upregulated Wnt/β-catenin signaling and reduced GSK3β mRNA expression; this could be one of the explanations accepted for the demonstrated decreased BACE1 and Aβ and the enhanced BDNF level. The results of Hamdan et al., 2022 partially supported our findings; they reported increased Wnt3a and β-catenin mRNA and decreased GSK3β expression with MH and PhM [12]. Studies investigating the impact of ZC on Wnt 3/β-catenin/GSK-3β signaling are limited; only one study conducted by Omran et al., (2022) was found, and in contrast to ours, they demonstrated decreased β-catenin content with ZC treatment, although the phosphorylated inactive GSK-3β content was increased [109].

In addition, LRP1 scavenges the harmful Aβ from the brain and is responsible for mediating key physiological activities of ApoE4 [57]. Furthermore, tau internalization and degradation are mediated by LRP1 [110]. However, ApoE4 competes and has a greater binding affinity for LRP1, halting LRP1-mediated tau uptake and Aβ clearance, resulting in its accumulation [36,110,111]. The results of this study revealed significantly higher ApoE4 levels and lower LRP1 levels in the AlCl_3_-administered group as opposed to the control, which support the previous reports of Hamdan et al. (2022) and Abu-Elfotuh et al. (2024) [12,72] and largely suggest the impaction of the ApoE4/LRP1 axis in the AlCl_3_-induced neurotoxic effect. The present study’s most significant discovery is that the ApoE4/LRP1 axis was significantly impacted by the combination of MH and ZC in conjunction with PhM. Studies investigating the effects of MH, ZC, or PhM on the ApoE4/LRP1 axis in the context of AD are scarce. Hamdan et al. (2022) reported similar results with MH and PhM [12]; however, herein, we further elucidate the effects of ZC on the ApoE4/LRP1 axis and its enhancing effect when combined with MH and PhM.

This study has potential limitations. First, we used only one concentration of MH and ZC without exploring dose–response relationships. Second, the ferrous load should have been assessed in brain tissues as iron-dependent lipid peroxidation is the driving force of ferroptotic cell death.

## 4. Materials and Methods

### 4.1. Animals

About 320–340 g of male Sprague Dawley rats were used in the experiments. Rats were purchased from the Nile Company in Cairo, Egypt, for use in the chemical and pharmaceutical industries. Rats were randomly distributed into cages (four rats per cage), with a standard temperature of 24 to 26 °C and a 12-h light/dark cycle. They were maintained on water, ad libitum, and a regular diet for five weeks.

### 4.2. Ethical Statement

In accordance with the National Institutes of Health’s Guide for the Care and Use of Laboratory Animals, the Ethics Committee of Al-Azhar University’s Faculty of Pharmacy (Girls) (No. 471/2024) authorized the study design (NIH Publications No. 8023, amended 1978).

### 4.3. Drugs and Chemicals

AlCl_3_.6H_2_O and morin hydrate (MH) were purchased from Sigma-Aldrich Chemical Co. (St. Louis, MO, USA, CAS numbers 7446-70-0 and 654055-01-3, respectively). Micronized zeolite clinoptilolite (ZC) with an average diameter of the particles of 5 microns was purchased from El-Nasr Co. for Chemical Industry, Cairo, Egypt, with product ID 605516305. Before use, 1 mL of distilled water was used to dissolve 20 mg of AlCl_3_, MH, and ZC. Then, phosphate buffer saline (PBS) was used in order to adjust the pH to 7.4 [112]. Additional reagents were of the best analytical caliber.

### 4.4. Experimental Design

As shown in Supplementary Figure S1, 70 male Dawley rats of 320–340 g (aged 6 months) were allocated at random to 7 groups (10/group):

In the control group, rats were given saline i.p. daily for 5 weeks.

In the Alzheimer’s disease (AD) group, rats were given AlCl_3_ (70 mg/kg, i.p.) every day for 5 weeks in order to establish an AD model [113].

In the AD + PhM group, rats were given the same dose of AlCl_3_ as the AD group and were subjected to PhM twice weekly (an ST on the first day and open field on the second one) [114].

In the AD + MH group, rats were given the same dose of AlCl_3_ as the AD group, along with administration of MH (20 mg/kg, p.o.) daily for 5 weeks [12].

In the AD + ZC group, rats were given the same dose of AlCl_3_ as the AD group, along with administration of ZC (100 mg/kg, p.o.) daily for 5 weeks [79,115].

In the AD + COMB group, rats were given the same dose of AlCl_3_ as the AD group along with daily administration of the combination treatment of MH and ZC for 5 weeks.

In the AD + COMB + PhM group, rats were given the same dose of AlCl_3_ as the AD group in addition to daily administration of the combination treatment of MH and ZC and were subjected to PhM twice weekly for 5 weeks.

The behavioral tests were conducted on the animals and documented 24 h after the last dosage was given. Twenty-four hours after the behavioral assessments were conducted, the animals were sacrificed by cervical translocation while sedated with 80 mg/kg of ketamine intraperitoneally. The brain tissues were obtained and soaked with PBS at pH 7.4, and tissue was sampled for biochemical analysis in addition to histological evaluation.

### 4.5. Physical and Mental Activities

PhM was performed through two behavioral tests: the swimming test (ST) [116] and the open field test. The tests were executed in accordance with Porsolt et al. [117] and Cunha and Masur [118].

### 4.6. Behavioral Tests for Evaluating the Extent of Neurodegeneration

The Y-Maze and Morris water maze response tests were carried out one day after the last dose to determine the extent of neurodegeneration following the five-week experiment. The Y-Maze spontaneous alternation test measured the spatial working type and short-term memory [119]. The technique was conducted according to Abu-Elfotuh et al., and the spontaneous alternation percentage (SAP%) was calculated [34]. The Morris water maze (MWM) test was performed to test spatial learning and memory. The technique was conducted according to Morris (64); the escape latency was recorded in addition to the amount of time spent in the target area.

### 4.7. Tissue Sampling

The brain tissues were collected and soaked with icy saline. For histological analysis, 4 brains per group were maintained in 10% solution of neutral buffered formalin. The remaining 6 brains in each group were immediately split into two halves that were the same size. The cerebral cortex and hippocampal tissues were isolated. A 10% homogenate (*w*/*v*) of 20 mM sodium phosphate buffer (SPB) was adjusted at pH 7.4. Afterward, the homogenate was centrifuged at 1800× *g* for 10 min at 4 °C. Biochemical assays were then performed for the supernatant. The initial section was homogenized in 50 mM (10% *w*/*v*) ice-cold Tris-HCl and 300 mM sucrose (pH 7.4). The second fraction, on the other hand, was stored at −80 °C for use in real-time PCR investigations. 

### 4.8. Histopathological Study

Brain samples were fixed in 10% of formaldehyde, and the tissues were then sectioned into pieces that were 5 μm thick after being fixed in paraffin. Haematoxylin and eosin (H&E) staining allowed for routine histological investigation, which was subsequently photographed as needed. The following pathological alterations were assessed in the cerebral cortex, hippocampus, and striatum of the various groups: inflammatory cellular infiltration, neuronal degeneration, and nuclear pyknosis. The following criteria were used to record the degree of histopathological changes in the various brain regions: +++ for severe, ++ for moderate, + for mild, and − for nil [120].

### 4.9. Immunohistochemical Study

Tissue slices that were about 5 μm thick were treated with 3% H_2_O_2_ for 20 min, then washed and incubated for an additional night at 4 degrees Celsius using anti-BDNF (Cat. # GB11559, Servicebio, Wuhan, Hubei, China). After giving the slices a PBS rinse, they were incubated for 20 min with a secondary antibody HRP Envision kit (DAKO). After another wash with PBS, the sections were treated with diaminobenzidine (DAB) for ten minutes. The samples were once again cleaned with PBS, dried, and clarified in xylene before being covered and slid for analysis and stained with haematoxylin. The standard protocol for regular histological examination was followed in carrying out all standard procedures [121]. The protocol for IHC analysis was as follows: for the purpose of calculating the mean area % of immunohistochemical expression of BDNF, six sample non-overlapping fields were chosen at random, and each tissue segment was scanned. Using Leica Microsystems GmbH’s (Wetzlar, Germany) full HD microscopic imaging technology and Leica Application software, (Imaje J 1.53t) these data were collected.

### 4.10. Biochemical Analysis

#### 4.10.1. Colorimetric Calculation of Oxidative Stress Markers

Colorimetric kits from Biodiagnostic, Cairo, Egypt, were used to measure the levels of malondialdehyde (MDA), superoxide dismutase (SOD), and total antioxidant capacity (TAC) in brain tissue homogenates.

#### 4.10.2. Fluorometric Determination of Brain Monoamines

Fluorometric analysis was used to measure the value of monoamines, namely, norepinephrine (NE), serotonin (5-HT), and dopamine (DA), using kits from Sigma-Aldrich Co. (St Louis, MO, USA). As previously mentioned, the fluorometric test was carried out [12].

#### 4.10.3. Enzyme-Linked Immunosorbent Assay (ELISA)

Tumor necrosis factor-α (TNF-α) and IL-1β concentrations were measured by the ELISA method utilizing Quantikine^®^ Rat TNF-α ELISA Kit (Cat. #RTA00, R&D Systems, Minneapolis, MN, USA) and Cusabio Life Science, Inc., Wuhan, China (Cat. #CSB-E08055r), respectively.

Furthermore, GPX4 [Cat. #MBS934198], amyloid precursor protein (APP) [Cat. #MBS150418], amyloid beta (Aβ) [Cat. # MBS726579], brain-derived neuroprotective factor (BDNF) [Cat. #MBS2019439], wingless type MMTV integration site family [Cat. #MBS9314182], member 3a (Wnt3a) [Cat. #MBS1607868], β-Catenin [Cat. #MBS261324], acetylcholinestrase (AChE) [Cat. #MBS725468], apoliprotein E4 (ApoE4) [Cat. #MBS263133], β-secretase enzyme (BACE1) [Cat. #MBS2886958], and low-density lipoprotein receptor-related protein 1 (LRP1) [Cat. #MBS723580] were also measured in brain tissues using ELISA kits from MyBioSource, Inc., San Diego, CA, USA, in compliance following the guidelines provided by the manufacturer. The Quantikine^®^ Rat TNF-α ELISA Kit (Cat. #RTA00, R&D Systems, Minneapolis, MN, USA) and Cusabio Life Science, Inc., Wuhan, China (Cat. #CSB-E08055r), were used to determine levels of IL-1β and TNF-α, respectively.

#### 4.10.4. Real-Time Quantitative Polymerase Chain Reaction (RT-qPCR)

The mRNA expression levels of Tau, nuclear factor kappa-B (NF-κβ), B-cell lymphoma 2 protein (Bcl-2), B-cell lymphoma protein 2 (Bcl-2)-associated X protein (Bax), caspase-1, pyrin domain–containing-3 (NLRP3), glycogen synthase kinase-3β (GSK3β), heme-oxygenase-1 (HO-1), nuclear factor erythroid 2–related factor 2 (Nrf2), toll-like receptor-4 (TLR4), Solute Carrier Family 7 Member 11 (SCL7A11), Acyl-CoA Synthetase Long Chain Family Member 4 (ACSL4), and the housekeeping gene (β-actin) in the brain were all measured using real-time quantitative polymerase chain reaction (RT-qPCR) utilizing Applied Biosystems step one plus instruments. Total RNA was isolated utilizing a Qiagen tissue extraction kit (Qiagen, Germantown, Maryland, USA). Utilizing a sensibly quick cDNA synthesis kit (Cat. No. BIO-65053), the mRNA that was obtained underwent reverse transcription. The following formula was used to determine the target genes’ relative expression: 2^−ΔΔCT^ [122]. The primer sets’ sequences can be seen in Table 2.

### 4.11. Statistical Analysis

The data are displayed as the mean ± SD of six experiments using GraphPad Prism 6 (ISI^®^, Fairfield, NJ, USA) software. The comparisons between the experimental groups were executed using one-way ANOVA followed by the Tukey–Kramer multiple comparisons test. *p* < 0.05 was considered as statistically significant.

## 5. Conclusions

**Figure 9:**
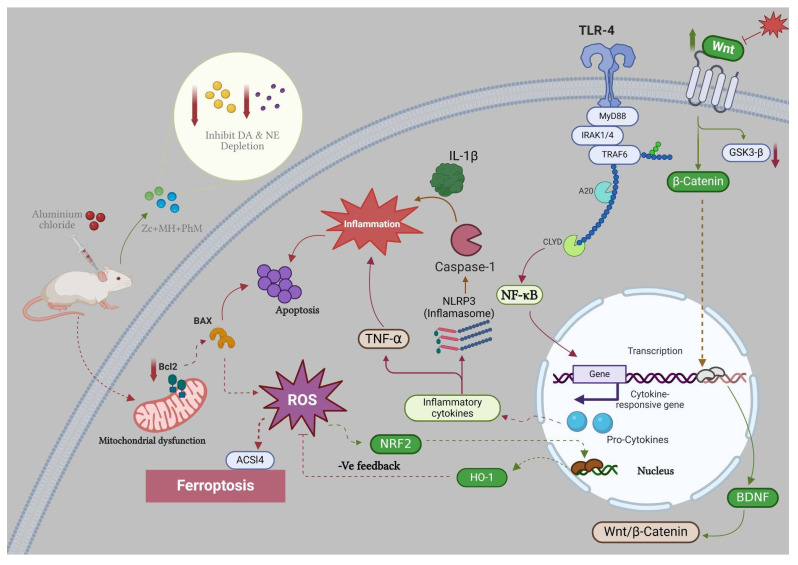
Graphical abstract. The modulatory impact of MH or/and ZC with or without physical and mental activities on the behavioral and biochemical modifications stimulated by AlCl_3_ in rats. The combination of MH and ZC alongside PhM significantly increased the TAC, SOD, Nrf2, HO-1, and GPX4 levels and decreased ACSL4 expression. Additionally, it downregulated TLR4/NLRP3/caspase-1 signaling, upregulated the Wnt/β-catenin signaling, and reduced the GSK3β mRNA expression.

For the first time, we documented the superior neuroprotective effect of combining MH and ZC with PhM as opposed to PhM, MH, ZC, or MH + ZC independently, demonstrating the effectiveness of the integrated multifaceted treatments in ameliorating AlCl_3_-induced AD features. This study is the first to point out the involvement of ferroptosis-Nrf2/inflammasomes cross-talk in the neuroprotection mechanism of MH/ZC plus phM against the AlCl_3_-mediated AD model. Additionally, by evaluating the impacts on Nrf2/HO-1/ACSL4/GPX4, TLR4/NF-κβ/NLRP3, APOE4/LRP1, and Wnt 3/β-catenin/GSK-3β signaling pathways, we have outlined the underlying molecular mechanisms pertinent to this mediated neuroprotective effect (Figure 9).

## Figures and Tables

**Table 1:** Neurotransmitter levels in the brain tissues.

Parameter	Control	AD	AD + PhM	AD + MH	AD + ZC	AD + COM	AD + COM + PhM
DA	71.22 ± 1.8	13.63 ± 1.8 ^a^	23.7 ± 2.4 ^ab^	40 ± 0.1 ^abc^	35.85 ± 2 ^abc^	49.87 ± 0.1 ^abcde^	58.12 ± 2 ^abcdef^
5-HT	11.82 ± 0.9	1.93 ± 0.1 ^a^	4.21 ± 0.4 ^ab^	5.95 ± 0.4 ^abc^	7.26 ± 0.5 ^abcd^	9.71 ± 0.5 ^abcde^	11.23 ± 0.8 ^bcdef^
NE	737.2 ± 4	176.6 ± 8 ^a^	241.3 ± 14 ^ab^	390.7 ± 14 ^abc^	441.4 ± 13 ^abcd^	533.5 ± 26 ^abcde^	635.4 ± 34 ^abcdef^
ACHE	11.75 ± 1.08	70.47 ± 0.8 ^a^	62.45 ± 1.8 ^ab^	44.83 ± 0.7 ^abcde^	35.87 ± 1.2 ^abcde^	22.42 ± 1.8 ^abce^	13.76 ± 1 ^bcd^

The data are displayed as means ± SD (*n* = 6) using one-way ANOVA and then Tukey–Kramer as the post hoc test. Significance a, with respect to the control group; significance b, with respect to the AD group; significance c, with respect to the AD + PhM group; significance d, with respect to the AD + MH group; significance e, with respect to the AD + ZC group. Significance f, with respect to the AD + COMB group. Significance: *p* < 0.05. AD, Alzheimer’s disease; COMB, combination therapy; MH, morin hydrate; PhM, physical and mental activity; ZC, zeolite clinoptilolite; 5-HT, serotonin; ACHE, acetylcholinesterase; DA, dopamine; NE, norepinephrine.

**Table 2:** List of primers.

Gene	Forward Primer	Reverse Primer	Accession No.
*NF-kβ*	5′-GGACAGCACCACCTACGATG-3′	5′-CTGGATCACTTCAATGGCCTC-3′	NM_001276711
*Bax*	5′-CACGTCTGCGGGGAGTCA-3′	5′-TAGGAAAGGAGGCCATCCCA-3′	NM_017059
*Bcl-2*	5′-CATCTCATGCCAAGGGGGAA-3′	5′-TATCCCACTCGTAGCCCCTC- 3′	NM_016993
*Caspase-1*	5′-GAACAAAGAAGGTGGCGCAT-3′	5′-GAGGTCAACATCAGCTCCGA-3′	NM_012762
*NLRP3*	5′-TGCATGCCGTATCTGGTTGT-3′	5′-ACCTCTTGCGAGGGTCTTTG-3′	NM_001191642
*GSK3β*	5′-AGCCTATATCCATTCCTTGG-3′	5′-CCTCGGACCAGCTGCTTT-3′	NM_032080
*HO-1*	5′-CACCAGCCACACAGCACTAC-3′	5′-CACCCACCCCTCAAAAGACA-3′	NM_012580
*Nrf-2*	5′-CTCTCTGGAGACGGCCATGACT-3′	5′-CTGGGCTGGGGACAGTGGTAGT-3′	NM_031789
*TLR4*	5′-TCAGCTTTGGTCAGTTGGCT-3′	5′-GTCCTTGACCCACTGCAAGA-3′	NM_019178
*Tau*	5′-TAGCTGACGAGGTGTCTGCC-3′	5′-ATTTGAAGGACTTGGGGAGG-3′	NM_017212
*ACSL4*	5′-TCCCTGGACTAGGACCGAAG-3′	5′-GGGGCGTCATAGCCTTTCTT-3′	NM_001431649
*β-actin*	5′-CCGTAAAGACCTCTATGCCA- 3′	5′-AAGAAAGGGTGTAAAACGCA- 3′	NM_031144

## Data Availability

The authors confirm that the data supporting the findings of this study are available within the article.

## Supplementary Materials

The following supporting information can be downloaded at: https://www.mdpi.com/article/10.3390/ijms26031260/s1.

## Abbreviations

AD;	Alzheimer’s disease
AlCl_3_	Aluminum chloride
MH	Morin hydrate
ZC	Zeolite clinoptilolite
PhM	Physical and mental activities
Aβ	Amyloid beta-peptide
BDNF	Brain derived neurotrophic factor
ACHE	Acetylcholinesterase
GSH	Reduced glutathione
HO-1	Heme oxygenase-1
Bax	B-cell lymphoma protein 2 (Bcl-2)-associated X protein
Bcl-2	B-cell lymphoma 2
DA	Dopamine
TNF-α	Tumor necrosis factor-α
ELISA	Enzyme-linked immunosorbent assay
GSK-3β	Glycogen synthase kinase-3β
H&E	Haematoxylin and eosin
NLRP3, NOD-, LRR-	Pyrin domain-containing protein 3
IL-1β	Interleukin-1β
NE	Norepinephrine
NF-κB	Nuclear factor-κappa B
Nrf2	Nuclear factor erythroid 2 related factor 2
Wnt3a	Wingless type MMTV integration site family, member 3a
