# Supplementary figures and images for: Targeting Ferroptosis/Nrf2 Pathway Ameliorates AlCl3-Induced Alzheimer’s Disease in Rats: Neuroprotective Effect of Morin Hydrate, Zeolite Clinoptilolite, and Physical Plus Mental Activities
Source: Int J Mol Sci. 2025 Jan 31;26(3):1260. doi: 10.3390/ijms26031260 (PMC11818523; doi:10.3390/ijms26031260)

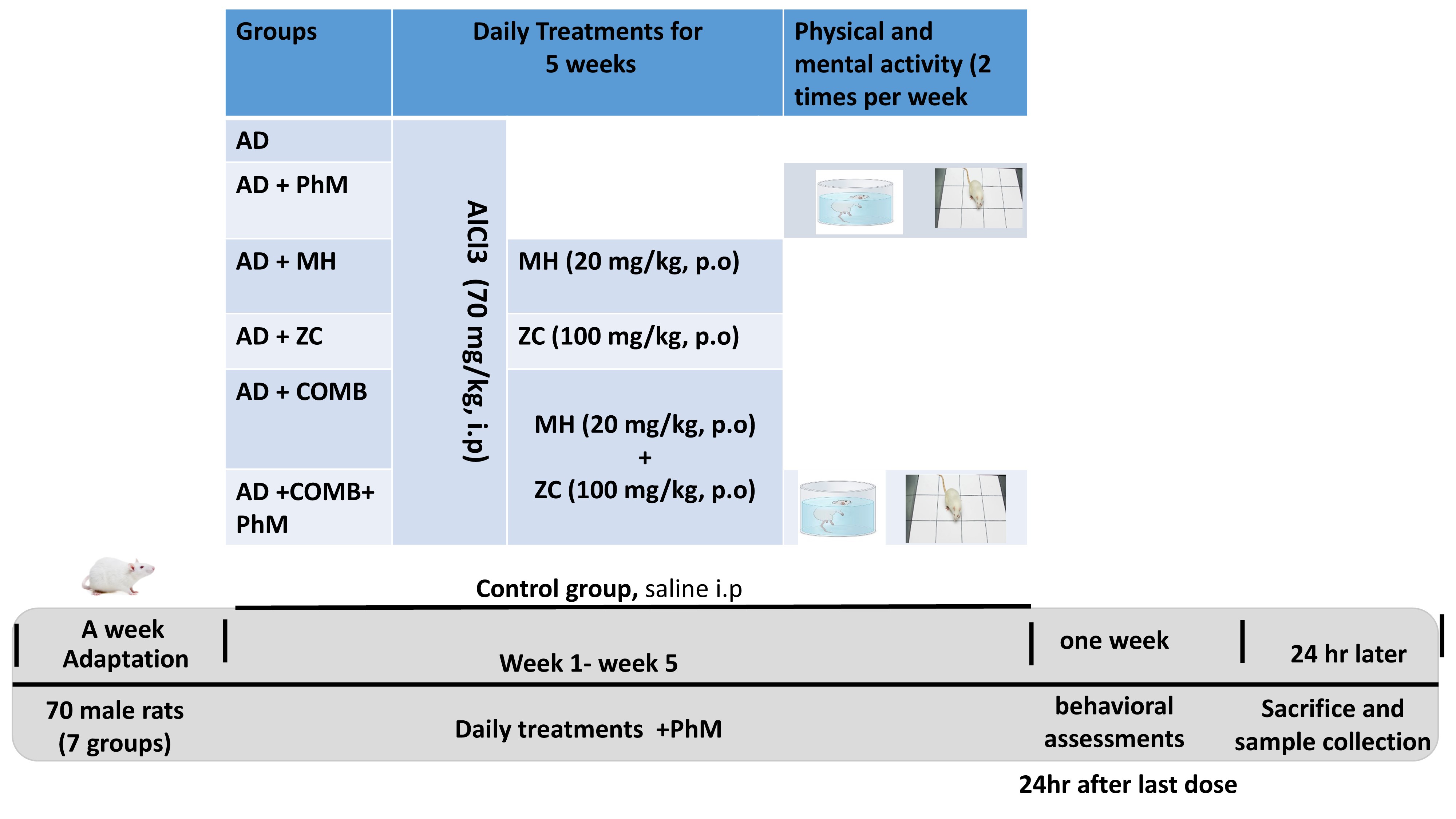

Supplement: Supplementary file 1 [file ijms-26-01260-s001.zip › ijms-3419842-supplementary.jpg]
